# A bioinformatics approach to systematically analyze the molecular patterns of monkeypox virus-host cell interactions

**DOI:** 10.1016/j.heliyon.2024.e30483

**Published:** 2024-04-29

**Authors:** Zhongxiang Tang, Ying Han, Yuting Meng, Jiani Li, Xiangjie Qiu, Ousman Bajinka, Guojun Wu, Yurong Tan

**Affiliations:** a Department of Medical Microbiology, Xiangya School of Medicine, Central South University, Changsha, 410078, Hunan, China; b China-Africa Research Center for Infectious Diseases, School of Basic Medical Sciences, Central South University, Changsha, 410078, Hunan, China; c Department of Stomatology, Center of Stomatology, Xiangya Hospital, Central South University, Changsha, Hunan, 410008, China

**Keywords:** Monkeypox, Transcriptome sequencing, Protein-protein interaction, IFIT2

## Abstract

Monkeypox has been spreading worldwide since May 2022, when the World Health Organization (WHO) declared the outbreak a “public health emergency of international concern.” The spread of monkeypox has posed a serious threat to the health of people around the world, but few studies have been conducted, and the molecular mechanism of monkeypox after infection remains unclear. We therefore implemented a transcriptome analysis to identify signaling pathways and biomarkers in monkeypox-infected cells to help understand monkeypox-host cell interactions. In this study, datasets GSE36854 and GSE11234 were obtained from GEO. Of these, 84 significantly different genes were identified in the dataset GSE36854, followed by KEGG, GO analysis protein-protein interaction (PPI) construction, and Hub gene extraction. We also analyzed the expression regulation of hub genes and screened for drugs targeting hub genes. The results showed that monkeypox-infected cells significantly activated the cellular immune response. The top 10 hub genes are IER3, IFIT2, IL11, ZC3H12A, EREG, IER2, NFKBIE, FST, IFIT1 and AREG. AP-26113 and itraconazole can be used to counteract the inhibitory effect of monkeypox on IFIT1 and IFIT2 and serve as candidate drugs for the treatment of monkeypox virus infection. IRF1 may also be a transcription factor of IFIT. Our results provide a new entry point for understanding how monkeypox virus interacts with its host.

Trial registration: N/A.

## 1 Introduction

Monkeypox is a viral zoonosis caused by monkeypox virus infection and used to occur mostly in West and Central Africa [1,2]. However, since May 2022, many countries and regions outside Africa have reported cases of monkeypox. There is no direct epidemiological link between this outbreak and the Central and West African regions [3]. The WHO declared the multinational outbreak a “public health emergency of international concern” on July 23, 2022 [4].

Monkeypox, like smallpox and vaccinia viruses, is a member of the orthopoxvirus family and is usually less severe than smallpox [5]. In general, monkeypox is a self-limiting disease, with symptoms lasting 2–4 weeks and a mortality rate of about 3%–6% [6]. Among them, the monkeypox clade from West Africa caused a mortality rate of about 3.6 %, while the monkeypox clade from the Congo Basin caused a mortality rate of up to 10.6 % [7]. Monkeypox usually incubates for 5–21 days, which is not infectious. Symptoms usually include fever, headache, lymphadenopathy, muscle soreness, and fatigue. Among them, lymphadenopathy is a prominent feature of monkeypox and can be distinguished from similar diseases such as chickenpox, measles, and smallpox [8]. Rashes usually appear within 3 days of onset, first on the face and then on the limbs (hands, legs, and feet). Oral mucosa, genitalia, conjunctiva, and cornea may also be present. Animal-to-human transmission is the main route of transmission through animal bites or direct contact with animal blood, body fluids, skin or mucosal lesions, etc., or improperly cooked animals infected with monkeypox virus. Human-to-human transmission includes respiratory droplets, direct contact, or sexual transmission [9,10]. Current evidence for suspected mother-to-child transmission is insufficient [11]. Some of the people affected by this outbreak are MSM (Men who have sex with men) [12]. Currently, researchers have reported cases of monkeypox in human-animal transmission [13] and cases of monkeypox combined with Sars-Cov-2 infection [14], This mixed transmission with animals and the superimposed transmission of Sars-Cov-2 will cause the accelerated spread of monkeypox and unpredictable results.

Given the seriousness of the monkeypox epidemic, in addition to the need to clarify the many causes of the outbreak and to find ways to limit the spread of monkeypox, there is also an urgent need to clarify the mechanisms by which monkeypox interacts with humans to cause severe disease. In this study, we obtained transcriptinal sequence data from monkeypox infected cells in Gene Expression Omnibus (GEO) and screened significantly different genes, analyzed the signaling pathway, expression regulation and metabolic pathway associated with significantly different genes. We also compared the difference between monkeypox, cowpox and vaccinia in changes in cell transcripts after infection.

## 2 Methods

### 2.1 Acquisition of sequencing data and differential gene analysis

To determine the effect of monkeypox infection on human cells at a transcriptional level, we downloaded sequence datasets GSE36854 and GSE11234 from the GEO Database [15]. The dataset GSE36854 was sequenced on Agilent's GPL4133. The dataset contains 8 samples, divided into 4 groups, with two samples in each group. The four groups simulated infection of HeLa cells with vaccinia virus strain IHD-W, vaccinia virus strain Brighton Red, monkeypox virus strain msf#6 and blank group, respectively [16]. The dataset GSE11234 is based on GPL6763, which contains many types of poxvirus [17]. We only extracted samples of HeLa cells infected with monkeypox for analysis of genetic alterations. We processed and visualized sequence data using R (R version 4.1.3) language in which the Limma package was used to screen for significantly differentially expressed genes [18]. We used | Log2FoldChange | ≥1 and the *p*-value <0.05 as screening criteria for significantly different genes.

### 2.2 Pathway enrichment and gene ontology analysis

Pathway enrichment and Gene ontology analysis for significantly different genes can effectively establish the intrinsic association among multiple genes. This in turn helps us understand cell signaling, gene regulatory networks, and the composition of biological systems [19,20]. Gene ontology (GO) analysis can be divided into three parts: Molecular function, biological process and cellular component, which can systematically describe the function, biological process and cellular localization of significantly different genes. We performed KEGG and GO analysis using the online Enrichr analysis platform, which is a comprehensive online tool for gene enrichment analysis that contains a large number of genomic annotation libraries for analysis [21]. KEGG 2021 was used as the pathway enrichment analysis database, and pathway differences of significantly different genes were compared among the three virus-infected HeLa cells. *P* < 0.05 was used as the “significance” criterion. Gene Set Enrichment Analysis (GSEA) was performed to identify pathways based on transcript sequences obtained from monkeypox viral infection [22].

### 2.3 Identification of transcription factors and miRNAs regulating DEGs

Expression regulation of significantly different genes is the underlying logic for significant changes in monkeypox. Therefore, elucidating the regulation of expression of significantly different genes will help us to find the key to the occurrence of monkeypox infection. It will also help us target key cellular-intrinsic gene links involved in the pathophysiological response to monkeypox infection. We analyzed significant differences in monkeypox-infected hela cells using the online analysis tool NetworkAnalyst 3.0 (https://www.networkanalyst.ca/) for gene expression and meta-analysis [23]. The ENCODE database (transcription factor and gene target data derived from ENCODE ChIP-seq data) was used to analyze the expression regulation relationship between transcription factor and DEGs [24]. The TarBase V8.0 database (comprehensive experimentally validated miRNA-gene interaction data collected from TarBase) was used to analyze the expression regulation relationship between miRNAs and DEGs [25,26].

### 2.4 Protein-protein interaction network analysis and hub gene extraction

The PPI network using the String (https://string-db.org, version: 11.5) database of significantly different genes for monkeypox infection was constructed to explore possible relationships among DEGs [27]. These include, but are not limited to, co-expression and physical relationship. We also used the Density of Maximum Neighborhood Component (DMNC) method in the Cytohubba plug-in (version:3.8.2) to extract the top 10 genes in this PPI as hub genes [28,29].

### 2.5 ENCODE data verify transcription factor binding

The Encode (https://www.encodeproject.org/) database was used to analyze the binding of IRF1 to the IFITs promoter, which was visualized with the WashU Epigenome Browser online tool (http://epigenomegateway.wustl.edu/browser/) [24,30].

### 2.6 Drug sensitivity and gene-disease association analysis

The Gene-disease Associations tool of network analysts in the analysis platform was used to analyze diseases involving significantly different genes based on the DisGeNET database. The curated literature on gene-disease association was collected from the DisGeNET database [31]. Expression data for IFIT1 and IFIT2 in the NCI-60 cell line drug database were downloaded from the CellMiner database. The Pearson correlation coefficient with 792 FDA-approved drugs in clinical trials was analyzed, and the results were screened and visualized (*p* < 0.05) [32].

## 3 Results

### 3.1 Screening for differentially expressed genes

**Fig. 1:**
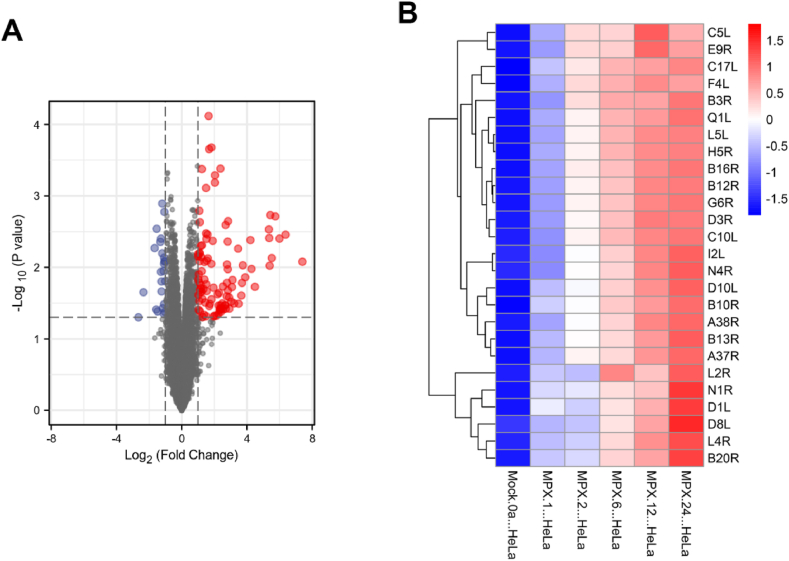
Screening for significantly different genes. A, volcanic plot of differential genes in monkeypox-infected HeLa cells in the GSE36854 dataset. B, heatmap of monkeypox early genes in monkeypox-infected HeLa cells in the GSE11234 data set.

By comparing expression profiles of monkeypox virus-infected and mock-infected HeLa cells in the GSE36854 dataset, we screened 126 significantly different genes, 24 of which were significantly downregulated and 102 significantly upregulated (Fig. 1A). In addition, we found that cowpox virus resulted in 390 significantly altered genes including 168 significantly upregulated genes and 222 significantly downregulated genes. Vaccinia virus resulted in 390 significant gene changes, including 131 significantly upregulated genes and 97 significantly downregulated genes. By comparing the expression profiles of monkeypox infection and mock infection in the GSE11234 dataset, we identified early viral genes expressed in HeLa cells after monkeypox infection (Fig. 1B). The dataset contains point-in-time information.

### 3.2 GO and pathway enrichment analysis

**Fig. 2:**
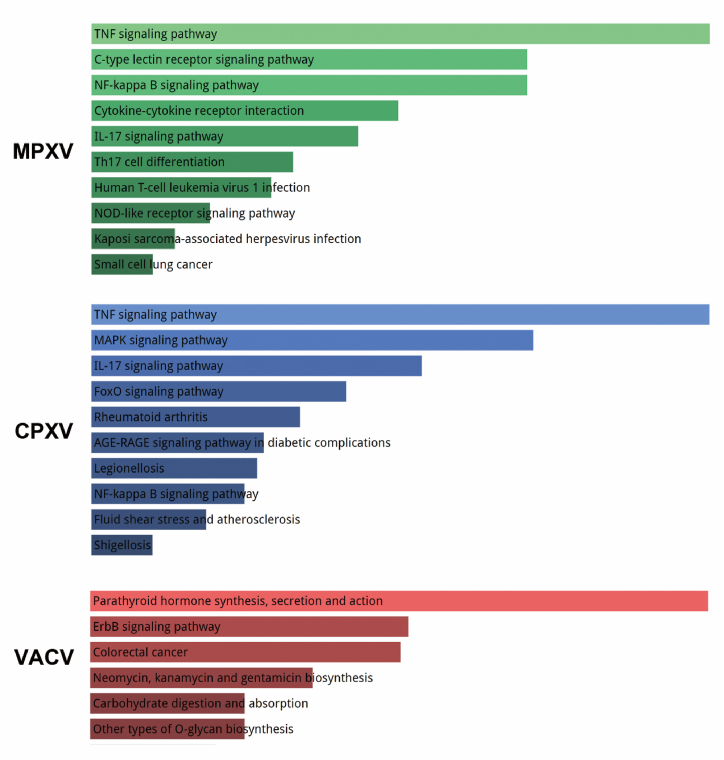
KEGG signaling pathway with significantly different gene enrichment produced by MPXV, CPCV and VACV in HeLa cells.

**Fig. 3:**
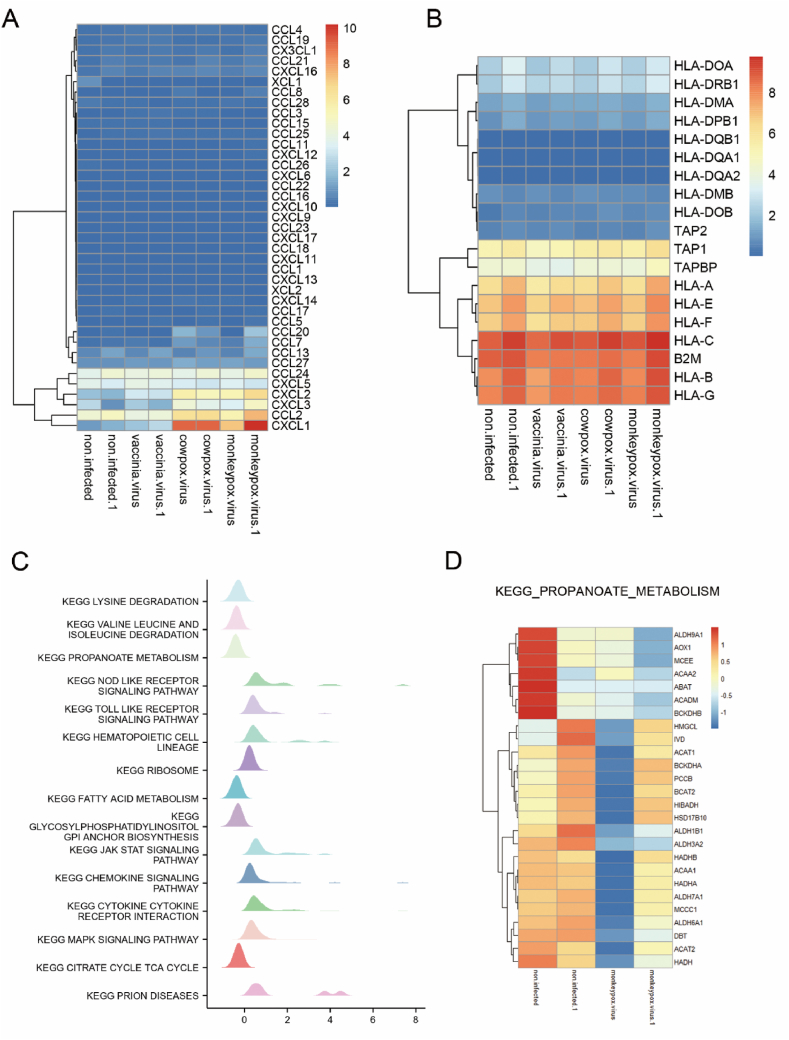
GSEA analysis of the signaling pathway in monkeypox-infected HeLa cells. A, heatmap of chemokines expression in HeLa cells infected with monkeypox, vaccinia and vaccinia. B, Heatmap of MHC molecule expression in monkeypox, vaccinia and vaccinia-infected HeLa cells. (C) Gene changes in HeLa cells infected with monkeypox. D, heatmap depicts the signal pathway changes in Propanoate metabolism.

The results showed that the signal pathways of monkeypox and vaccinia were mainly enriched in the TNF signaling pathway, IL-17 signaling pathway and NF-kappaB signaling pathway. However, vaccinia altered signaling pathways are mainly enriched in parathyroid hormone synthesis, secretion and action, ErbB signaling pathway, colorectal cancer, Neomycin, kanamycin and gentamicin biosynthesis, carbohydrate digestion and absorption, and other types of O-glycan biosynthesis (Fig. 2). It is suggested that monkeypox and vaccinia may cause more stress and pathology than vaccina. GO analysis also showed that monkeypox infection activated a variety of chemotactic signaling pathways. Table 1 summarizes the top 10 terms in the class of biological processes, molecular functions, and cellular components involved in differentially expressed genes. To determine key chemotactic responses after monkeypox infection, we analyzed changes in chemokines and MHC molecules after infection in three viruses. The results showed that CXCL1 was significantly activated after vaccinia and monkeypox infection (Fig. 3B), however, MHC molecules were largely unchanged after viral infection (Fig. 3A), suggesting that CXCL1 plays a key role in controlling chemotaxis after monkeypox infection.

**Table 1:** Biological processes, molecular functions and cellular components involved in differentially expressed genes.

Category	GO ID	Term	P-value	Genes
GO Biological Process	GO:0071345	cellular response to cytokine stimulus	1.59E-09	EGR1; IL11; IL4R; EDN2; LIF; CXCL1; IL1RAP; IFIT1; PTGS2; CXCL2; IL27RA; EREG; ZFP36; IL6; ZC3H12A; SH2B3; STX1A; GBP3
	GO:0019221	cytokine-mediated signaling pathway	1.39E-08	EGR1; IL11; IL4R; EDN2; LIF; CXCL1; IL1RAP; IFIT1; PTGS2; CXCL2; IL27RA; IFIT2; EREG; NFKBIA; IL6; TNFRSF25; SH2B3; STX1A; BIRC3
	GO:0007176	regulation of epidermal growth factor-activated receptor activity	6.88E-07	ERRFI1; CBLB; AREG; ADRA2A; EREG
	GO:0042059	negative regulation of epidermal growth factor receptor signaling pathway	8.49E-06	ERRFI1; SPRY2; CBLB; AREG; EREG
	GO:0043065	positive regulation of apoptotic process	2.93E-05	VAV3; IL6; ZC3H12A; ARHGEF16; HMGA2; ARHGEF4; BMF; PHLDA1; DUSP6; IFIT2
	GO:0045741	positive regulation of epidermal growth factor-activated receptor activity	3.98E-05	AREG; ADRA2A; EREG
	GO:0030219	megakaryocyte differentiation	5.28E-05	IL11; CDKN2B; SH2B3
	GO:0045944	positive regulation of transcription by RNA polymerase II	6.20E-05	LMO1; EGR1; IL11; EGR2; CDKN2B; LIF; NR2F1; HMGA2; FOXO4; KLF2; FOSL1; NFKBIA; IL6; ZC3H12A; NAMPT; MAFF; IER2
	GO:0042981	regulation of apoptotic process	7.43E-05	VAV3; PLK3; ARHGEF16; HMGA2; DUSP6; IFIT2; NFKBIA; IL6; PROC; ARHGEF4; SPRY2; BMF; TNFRSF25; PHLDA1; BIRC3
	GO:0043068	positive regulation of programmed cell death	8.96E-05	VAV3; IL6; ARHGEF16; HMGA2; ARHGEF4; BMF; PHLDA1; DUSP6; IFIT2
GO Molecular functions	GO:0005126	cytokine receptor binding	4.79E-06	IL11; IL6; SPRED1; LIF; IL1RAP; SH2B3; IL27RA
	GO:0008083	growth factor activity	1.93E-05	IL11; IL6; LIF; CXCL1; AREG; EREG
	GO:0070851	growth factor receptor binding	5.61E-05	IL11; IL6; IL1RAP; AREG; IL27RA; EREG
	GO:0019901	protein kinase binding	3.87E-04	ERRFI1; ZFP36; SPRED1; CDKN2B; KCNQ1; SPRY2; SH2B3; DUSP6; ADRA2A; MAP2K6; RELB
	GO:0048018	receptor ligand activity	7.78E-04	IL11; IL6; EDN2; GDF15; LIF; CXCL1; AREG; EREG
	GO:0005125	cytokine activity	8.37E-04	IL11; IL6; GDF15; LIF; CXCL1; CXCL2
	GO:0030291	protein serine/threonine kinase inhibitor activity	8.96E-04	SPRED1; CDKN2B; SPRY2
	GO:0004860	protein kinase inhibitor activity	0.002563656	CDKN1C; SPRED1; SPRY2
	GO:0019900	kinase binding	0.002739834	ERRFI1; ZFP36; SPRED1; CDKN2B; SPRY2; STX1A; ADRA2A; MAP2K6; RELB
	GO:0045236	CXCR chemokine receptor binding	0.005111625	CXCL1; CXCL2
GO cellular component	GO:0030669	clathrin-coated endocytic vesicle membrane	0.009659115	AREG; LDLR; EREG
	GO:0045334	clathrin-coated endocytic vesicle	0.016926667	AREG; LDLR; EREG
	GO:0030665	clathrin-coated vesicle membrane	0.019680367	AREG; LDLR; EREG
	GO:0005587	collagen type IV trimer	0.037504666	COL4A4
	GO:1904724	tertiary granule lumen	0.047750884	CXCL1; METTL7A
	GO:0042406	extrinsic component of endoplasmic reticulum membrane	0.049693463	ZC3H12A

### 3.3 GSEA analysis

To more precisely characterize the genetic changes in cells following monkeypox infection, we performed GSEA analysis. We found that monkeypox viral infection significantly activated intracellular pattern recognition receptor pathways, such as NOD and TOLL, consistent with our KEGG and GO analyses of significantly different genes. Monkeypox virus infection also significantly reduced metabolic levels within cells, including Lysine degradation, Valine-Leucine and Isoleucine degradation, Propanoate metabolism, Fatty acid metabolism, Glycosylphosphatidylinositol-GPI-Anxhor biosynthesis and Citrate cycle-TCA cycle (Fig. 3C). We compared the differences in all genes of the Propanoate pathway before and after infection and found that monkeypox viral infection inhibited the expression of all genes of this pathway (Fig. 3D). This may be due to the hijacking of cellular metabolites after infection as a substrate for viral replication.

### 3.4 Identification of disease associations

**Fig. 4:**
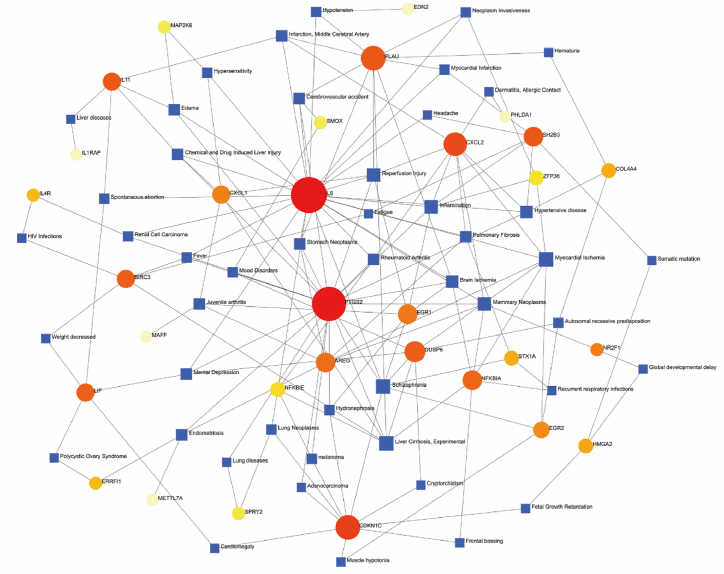
**DEGs and disease networks.** Circular nodes represent genes, and square nodes represent disease types.

To clarify the relationship between disease and the significantly different genes after viral infection with monkeypox, we used the Network Analysts' Gene-Disease Associations tool. The results showed that Liver Cirrhosis, Schizophrenia, Myocardial Ischemia, Inflammation, Reperfusion Injury, Mammary Neoplasms, Hypertensive Disease, Brain Ischemia, Mental Depression and Juvenile Arthritis were the 10 diseases with the highest degree of association, and IL6 and PTGS2 were the two genes with the highest degree of association with the diseases (Fig. 4).

### 3.5 Monkeypox-associated significantly different gene interactions and hub extraction

**Fig. 5:**
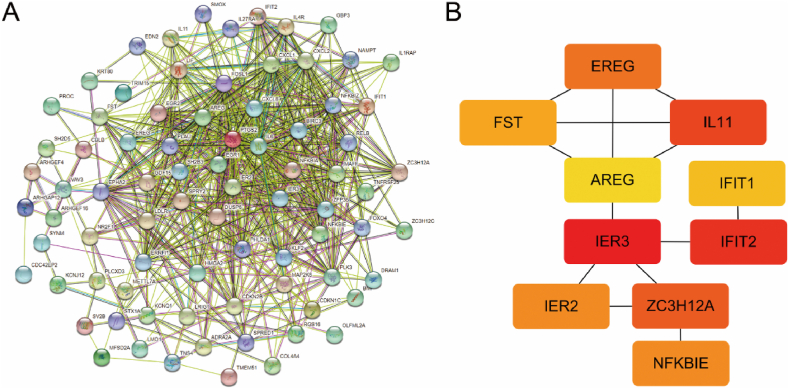
Shows the PPI network for DEGs. A, PPI networks of significantly different genes after monkeypox infection of HeLa cells were generated using the STRING database; B, the top 10 hub genes were extracted by the Density of Maximum Neighborhood Component method in Cytoscape cytohubba plug-in.

We constructed the interaction network of significantly different genes using string databases (Fig. 5A), and extracted the hub genes by DMNC method of SYTOHUBBA in cytoscape. The top 10 hub genes were IER3, IFIT2, IL11, ZC3H12A, Ereg, IER2, NFKBIE, FST, IFIT1 and AREG (Table 2,Fig. 5B).

**Table 2:** The changes of 10 hub genes after monkeypox infection.

Gene	logFC	P.Value
IFIT2	−1.56594	0.038802
IFIT1	−1.2736	0.01158
IL11	2.777671	0.009122
AREG	2.840347	0.002257
EREG	2.689366	0.002555
IER3	2.815185	0.004363
IER2	1.0182	0.014747
FST	1.390166	0.008395
ZC3H12A	2.289584	0.022705
NFKBIE	1.11534	0.002333

### 3.6 Verification of hub gene expression regulated by monkeypox virus

**Fig. 6:**
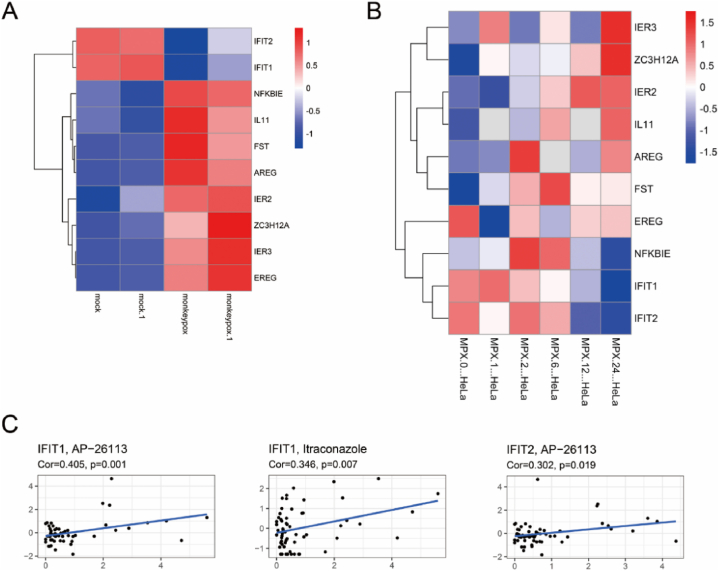
**Validation of hub genes.** A, heatmap of the 10 hub genes in the GSE36854 dataset; B, heat map of the 10 hub genes in the GSE11234 dataset; C, drug sensitivity assessment for IFIT1 and IFIT2.

In order to verify the effect of monkeypox virus on the expression of the 10 hub genes, we obtained the data set of monkeypox infected HeLa in GEO (GSE36854 and GSE11234). As shown in Fig. 6A–B, monkeypox virus significantly affected the expression of the 10 hub genes in the GSE11234 dataset, notably inhibiting IFIT1 and IFIT2 expression. Expression data for IFIT1 and IFIT2 in the NCI-60 cell line drug database were downloaded from the CellMiner database, and Pearson correlation coefficient with 792 FDA-approved drugs in clinical trials was analyzed. Results showed AP-26113 targeting IFIT1 and IFIT2 and Itraconazole targeting IFIT1 (Fig. 6C).

### 3.7 Hub genes regulatory network

**Fig. 7:**
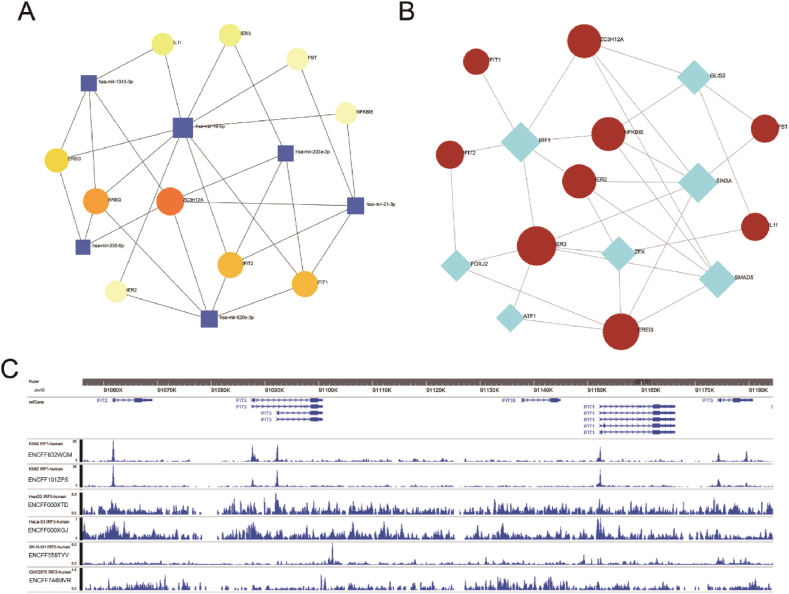
The expression regulatory network of hub genes. A, interaction network between hub genes and transcription factors based on NetworkAnalyst analysis; B, interaction network of hub genes with miRNAs based on NetworkAnalyst analysis; C, chip-seq sequencing data for IRF1 in the NCODE database on DNA binding strength of IFIT genes.

Genetic expression is mainly regulated by transcription factors and miRNAs.To explore the relationship between hub genes and regulatory molecules, we used the online network analyst tool to deconstruct the core relationships of hub genes with key transcription factors and miRNAs. As shown in Fig. 7A, the regulatory network of transcription factors with significantly different genes contains IRF1, SIN3A, GLIS2, Smad5, ZFX, FOXJ2, and ATF1 in turn. As shown in Fig. 7B, the regulatory network of miRNAs with significantly different genes are miR-16-5p, miR-21-3P, miR-520c-3p, miR-1343-3p, miR-203-3p and miR-335-5p. Given that IRF1 is the transcription factor directly interacting with both IFIT2 and IFIT1 in the regulatory network, we analyzed chip-seq data for IRF1 using the ENCODE database, which showed the presence of strong IRF1 binding intensity on chromosomes of IFIT family genes (Fig. 7C).

## 4 Discussion

As monkeypox continues to spread around the world, it poses a major public health problem worldwide. For researchers, clarifying the interaction between monkeypox and its host will be a prerequisite for dealing with monkeypox. Fortunately, there is sequencing data on monkeypox virus-infected cell models in the GEO data, which provides strong support for our study. We obtained two GEO sequencing datasets of monkeypox-infected HeLa, GSE36854, and GSE11234, and screened 84 genes that were significantly differentially expressed (excluding histone genes).

Early viral genes are extremely important for viral infection, survival, replication and spread, and are also involved in regulating host immunity. We screened 26 possible early viral genes including D1L. Moreover, after obtaining sequence data from many monkeypox samples, we found that D1L gene reported in 2022 epidemic have multiple site mutations compared to the D1L gene reported in the UK in 2018. Given that D1L is the Ankyrin repeat protein encoded by monkeypox virus, we speculate that this may be the cause of monkeypox becoming more adapted to human host and human-to-human transmission.

Signal pathway analysis can effectively reflect the internal body in real changes after external stimulation [33]. We found that these DEGs were involved in TNF signaling pathway, C-type lectin receptor signaling pathway, NF-kappa B signaling pathway, Cytokine-cytokine receptor interaction, IL-17 signaling pathway, Th17 cell differentiation, Human T-cell leukemia virus infection, NOD-like receptor signaling pathway, Kaposi sarcoma-associated herpesvirus infection and Small cell lung cancer. These results suggest that monkeypox infection can activate the body's strong immune response, produce an inflammatory response, and ultimately lead to pathological changes in the body.

Medical complications such as fever, rash, lymphadenopathy, pneumonia, encephalitis, eye-threatening keratitis, and subsequent bacterial infections may occur during monkeypox [6]. Different diseases can be linked by their intrinsic genetic similarity [34]. We therefore performed genetic disease (GD) analysis to predict the association of monkeypox DEGs with different diseases, and the results showed that monkeypox infection may cause liver cirrhosis, myocardial ischemia, inflammation, reperfusion injury, mammary neoplasms, hypertensive disease, brain ischemia, and juvenile arthritis. This is consistent with the results of complications and sequelae of monkeypox. Additionally, my results show that monkeypox infection is associated with schizophrenia and mental depression, which supports the conclusion that monkeypox infection can lead to psychiatric disorders, such as anxiety, depression, and depression responses [35,36]. Prostaglandin-endoperoxide synthase 2 (PTGS2), also known as cyclooxygenase 2 (COX-2), is the rate-limiting enzyme in the production of prostaglandins (PGs), plays an important role in various tumors and is also an important marker of iron death signaling pathway [37,38]. PTGS2 plays an important role in the infection process of many viruses. Respiratory syncytial virus (RSV) and parafenfluenza virus infection lead to upregulation of PTGS2 expression in airway bronchioles and bronchial epithelial cells and macrophages [39]. PTGS2 also promotes dengue and Sapovirus replication [40,41]. All these indicate that PTGS2 can be used as a potential target for antiviral drug development. The role of PTGS2 in the process of monkeypox infection has not been reported, but given the degree of association between PTGS2 and many diseases, it is reasonable to speculate that monkeypox virus regulates the pathological process by regulating PTGS2.

We extracted the hub genes of monkeypox-infected HeLa cells including IER3,IFIT2,IL11,ZC3H12A,EREG,IER2,NFKBIE,FST, IFIT1 and AREG. The expression of the early response gene immediate early response 3 (IER3), formerly known as IEX-1, is induced by a wide variety of stimuli, such as growth factors, cytokines, ionizing radiation, viral infection, and other types of cellular stress [42]. IFIT genes are usually silent or expressed at very low constitutive levels. The induction of IFIT transcription is triggered by a number of stimuli, usually in the context of viral and bacterial infections [43]. IFIT is usually induced by Type I and Type III interferons, with much weaker IFIT induction by Type II Interferons [44,45]. RNA viruses such as RSV, influenza virus, west Nile virus and vesicular stomatitis viruses are then exposed to TLRs, RLRs, and NLRS, resulting in large amounts of IFIT induction in the cell. DNA viruses (e.g. Human herpesvirus 1 and Cytomegalovirus) that activate DNA sensors or cyclic GMP-AMP synthase [cGAS] also induce large amounts of IFIT expression [[46], [47], [48]]. IFIT1 and IFIT2 can inhibit mRNA translation initiation by binding to multi-subunit eukaryotic translation initiation factor 3(eIF3) and interfering with the assembly of the pre-initiation complex (consisting of the 40S ribosomal subunit, EIF3, eIF2/GTP/Met-tRNAi, and EIF4F) [[49], [50], [51], [52], [53], [54]]. Specifically, IFIT2 inhibits West Nile virus and rabies replication in the central nervous system. In addition, IFIT2 protected mice from lesions caused by vesicular stomatitis virus and Sendai respiratory virus (SeV) [[55], [56], [57]]. Similarly, IFIT1 is an effector molecule that limits viral translation, and limits infection of viruses lacking RNA 2′-O methylation by binding to mRNAs lacking RNA 2′-o methylation [[58], [59], [60], [61]]. Given IFIT's positive role in antiviruses, viruses have evolved several strategies to evade IFIT's antiviral capacity [62,63]. Unlike other viruses, our results showed that monkeypox virus could significantly inhibit IFIT1 and IFIT2 expression in HeLa cells. It may be that both have evolved some mechanism to regulate IFIT expression and escape IFIT's antiviral effect.

The regulation of key genes by transcription factors and miRNAs after viral infection is very important to the pathological process. We found that the main transcription factors regulating hub genes were IRF1, SIN3A, GLIS2, Smad5, ZFX, FOXJ2, and ATF1, and miRNAs were hsa-mir-16-5p, hsa-mir -21-3p, hsa-mir −520c-3p, hsa-mir -1343-3p, hsa-mir -203-3p, and hsa-mir-335-5p. Although the major players in viral-activated signaling pathways are IRF-3 or IRF-7, IRF-1 may also be a transcription factor associated with activation in some cells [64,65]. IFIT2 and IFIT1 were shown to be potentially regulated by IRF1 from our results, which were validated in Chip-seq data for IRF1 in ENCODE data. Moreover, these miRNA functions are virus-related, such as hsa-mir-520c-3p, which can be up-regulated by HBV and drive invasion and migration of liver cancer cells [66] and hsa-mir-16-5p, which is an essential miRNA during *E. histolytica* infection and is also significantly induced by HSV and HIV [[67], [68], [69]]. Hsa-mir-21-3p may promote IAV replication by inhibiting HDAC8 [70]. There is no current report on monkeypox virus-related miRNAs, but given the important role of miRNAs throughout viral infection, we have reason to believe that miRNAs will be a hot spot in future monkeypox studies.

There is currently no effective treatment for monkeypox, and several antiviral drugs are thought to be effective against monkeypox. However, these drugs used to treat smallpox virus require scientific evaluation for the treatment of monkeypox [71]. Tecovirimat (TPOXX) is considered the most promising drug by targeting and inhibiting the activity of the orthopoxvirus protein VP37 (a highly conserved gene encoded by all members of the orthopoxvirus genus), and blocking their interaction with cellular Rab9 GTPase and Tip47, thereby preventing viral particle formation and inhibiting viral transmission within an infected host [5,72]. We performed drug sensitivity analyses using the CellMiner database and found that AP-26113 (Brigatinib), a tyrosine kinase receptor inhibitor and antitumor drug, significantly promoted IFIT1 and IFIT2 expression for the treatment of some forms of advanced non-small cell lung carcinoma [73]. But there have been no reports of AP-26113 being used as an antiviral drug. In addition, Itraconazole, a broad-spectrum triazole fungicide, which is also widely used in the fight against influenza and HIV, significantly promotes IFIT2 expression [74,75]. Because Itraconazole is also highly active against SARS-CoV-2 *in vitro*, it has also been used in combination with other drugs to treat Covid-19 infections [76,77]. There are no reports of Itraconazole against monkeypox, but based on its broad spectrum of antibacterial and antiviral effects, Itraconazole holds promise for the treatment of monkeypox infection in combination with other drugs.

It should be noted, however, that there are some shortcomings in this study. First of all, we believe that early monkeypox virus genetic code products such as D1L alter the cellular immune response pattern by interacting with host proteins or genes, suppressing host immunity and accelerating virus replication, and D1L mutations are possible reasons why monkeypox virus is becoming more adaptable to human and human-to-human transmission. However, we did not conduct relevant experiments due to the continuing difficulties in obtaining live monkeypox virus particles and the limited laboratory grade requirements for monkeypox research. Secondly, in terms of pathogenesis and drug prevention and control, we found a lot of clues, but no relevant verification. So there must be some mechanism or theory to explore.

## 5 Conclusion

Monkeypox virus significantly inhibited IFIT1 and IFIT2 antiviral genes and reduced a variety of cell-intrinsic metabolic activities. AP-26113 and Itraconazole promoting IFIT1 and IFIT2 expression may be used as novel candidates for the treatment of monkeypox virus infection.

## Declarations

**Ethical approval and consent to participate:** N/A.

**Consent for publication:** N/A.

## Data availability statement

The datasets presented in this study can be found in online repositories. The names of the repository/repositories and accession number(s) can be found in the article/supplementary material.

## Funding

This work was supported by the 10.13039/501100002858China Postdoctoral Science Foundation [GZC20233171].

## CRediT authorship contribution statement

**Zhongxiang Tang:** Visualization, Validation, Methodology, Investigation, Formal analysis, Data curation. **Ying Han:** Methodology, Formal analysis. **Yuting Meng:** Writing – review & editing, Validation, Supervision, Resources. **Jiani Li:** Writing – review & editing, Supervision, Resources. **Xiangjie Qiu:** Writing – review & editing, Validation, Resources. **Ousman Bajinka:** Writing – review & editing, Validation. **Guojun Wu:** Writing – review & editing, Validation, Resources. **Yurong Tan:** Writing – review & editing, Project administration, Funding acquisition, Conceptualization.

## Declaration of competing interest

The authors declare that they have no known competing financial interests or personal relationships that could have appeared to influence the work reported in this paper.
